# Think-Aloud Testing of a Novel Safer Drinking App for College Students During COVID-19: Usability Study

**DOI:** 10.2196/32716

**Published:** 2022-02-17

**Authors:** Jessica Gomez Smith, Nour Sami Alamiri, Grace Biegger, Christina Frederick, Jennifer P Halbert, Karen S Ingersoll

**Affiliations:** 1 Center for Behavioral Health & Technology Department of Psychiatry and Neurobehavioral Sciences University of Virginia School of Medicine Charlottesville, VA United States; 2 Center for Addiction and Prevention Research Department of Public Health Sciences University of Virginia School of Medicine Charlottesville, VA United States

**Keywords:** app development, college binge drinking, think aloud testing, formative research, mobile phone

## Abstract

**Background:**

Hazardous alcohol consumption, and binge drinking in particular, continues to be common among college students, posing the greatest risk for their health and safety. Despite widespread exposure to evidence-based preventive interventions among US undergraduates, only modest and temporary effects on risky drinking occur. Formative studies have demonstrated that students want a more engaging intervention tool for risky drinking that can be used *just in time*.

**Objective:**

The purpose of this study is to test the appeal, relevance, and perceived utility of a draft mobile app for safer student drinking at a public university in Virginia.

**Methods:**

Undergraduate student participants tested the draft mobile app via a web-based prototype that tailors to individual feedback with hot spots that responded to their taps to mimic app functionality. They narrated their impressions, navigation, and comments in a standardized think-aloud procedure. After each round of think-aloud interviews, researchers debriefed the investigators and developers to discuss findings and brainstorm app modifications.

**Results:**

Minor changes to the functionality and aesthetics would improve usability of the app (eg, option for light mode in app settings). Student testers recommended tailoring the app to the needs of college students and to aspects of the local university’s drinking culture.

**Conclusions:**

Findings from this study will be synthesized with information gained from other formative work to determine the final app features. We will test the app in a pilot randomized trial to assess app use and the impact of the app on college student drinking behavior over several months.

## Introduction

### Binge Drinking

Binge drinking, defined as consuming 5 (for men) or 4 (for women) standard drinks within approximately 2 hours on a single occasion [1], continues to be a public health concern, particularly among college students [1]. In this millennium, there has been an increase of over 200% in alcohol-related deaths and a 25% increase in alcohol overdose–related hospitalizations in the United States [2]. Binge drinking can result in short-term effects such as poor academic performance, but more seriously, it can lead to detrimental health outcomes such as alcohol use disorder and other health risks [3]. College binge drinking habits can predict the course of alcohol dependence and problems over a lifetime [4,5]. Developing effective interventions to reduce binge drinking among college students has the potential to improve lifetime drinking outcomes and prevent alcohol-related health and psychosocial problems among college students across the United States.

### Alcohol Consumption Among Undergraduates

Undergraduate students are commonly exposed to evidence-based binge drinking preventive and clinical interventions that aim to promote drinking safety [6-8]. An example of a common intervention across universities in the United States is the Brief Alcohol Screening and Intervention for College Students program, which provides a harm reduction intervention that uses personalized normative feedback and structured prompts to guide behavior change for students who have risky drinking habits or alcohol-related consequences [9]. Despite universities continually improving alcohol programming, these efforts yield minimal change and rates of dangerous alcohol consumption have not changed significantly; worse, the effects of these programs diminish over time [10-13]. Therefore, novel interventions that engage students and promote lower risk drinking behavior by college students are still needed. A tailored mobile app for students could provide personalized alcohol safety information in real time, either before or during a drinking episode.

### This Study

With extensive student involvement, and based on findings from other formative work [14-16], we designed a draft mobile app for safer student drinking. The aim of this study is to determine college students’ perspectives on the appeal, relevance, and perceived utility of the draft app.

## Methods

### Basis of App Features and Functions

The setting was a public university in Virginia with extensive alcohol programming, alcohol interventions, and student involvement in governance, which has experienced several high-profile alcohol-related injuries and deaths of students in the past decades. We sought to identify new ways to reduce hazardous drinking among college students. We conducted 2 studies before this one. First, we conducted in-depth interviews and focus groups with students and university stakeholders to understand the culture of the university, the role of drinking in student and alumni life, the history and impact of various alcohol interventions, the continuing ubiquity of binge drinking despite deployment of numerous interventions, and students’ impressions of the impact of alcohol interventions to which they had been exposed (Alamiri, N, unpublished data, January 2022). Second, we conducted a review of public social media posts to understand how alcohol use and its consequences at the university were documented on the web by students and bystanders [17]. These formative studies demonstrated that (1) risky drinking is a prominent feature of student life, (2) most students have participated in risky drinking behavior, (3) students report minimal change in their own drinking in response to existing alcohol programming, (4) mobile app use by students is ubiquitous, and (5) students wanted flexible intervention tools that are accessible during drinking episodes (their own or when witnessing excessive drinking by their peers). Students requested a novel intervention with evidence-based and student-preferred features and functions. An estimated blood alcohol content (eBAC) calculator (to estimate the percentage of alcohol in a person’s bloodstream [15]) was the feature students were most interested in when the concept was presented during the focus groups and surveys. Overall, the formative findings indicated that novel interventions must be highly relevant to drinking behaviors and be usable during drinking.

### App Prototype

**Figure 1:**
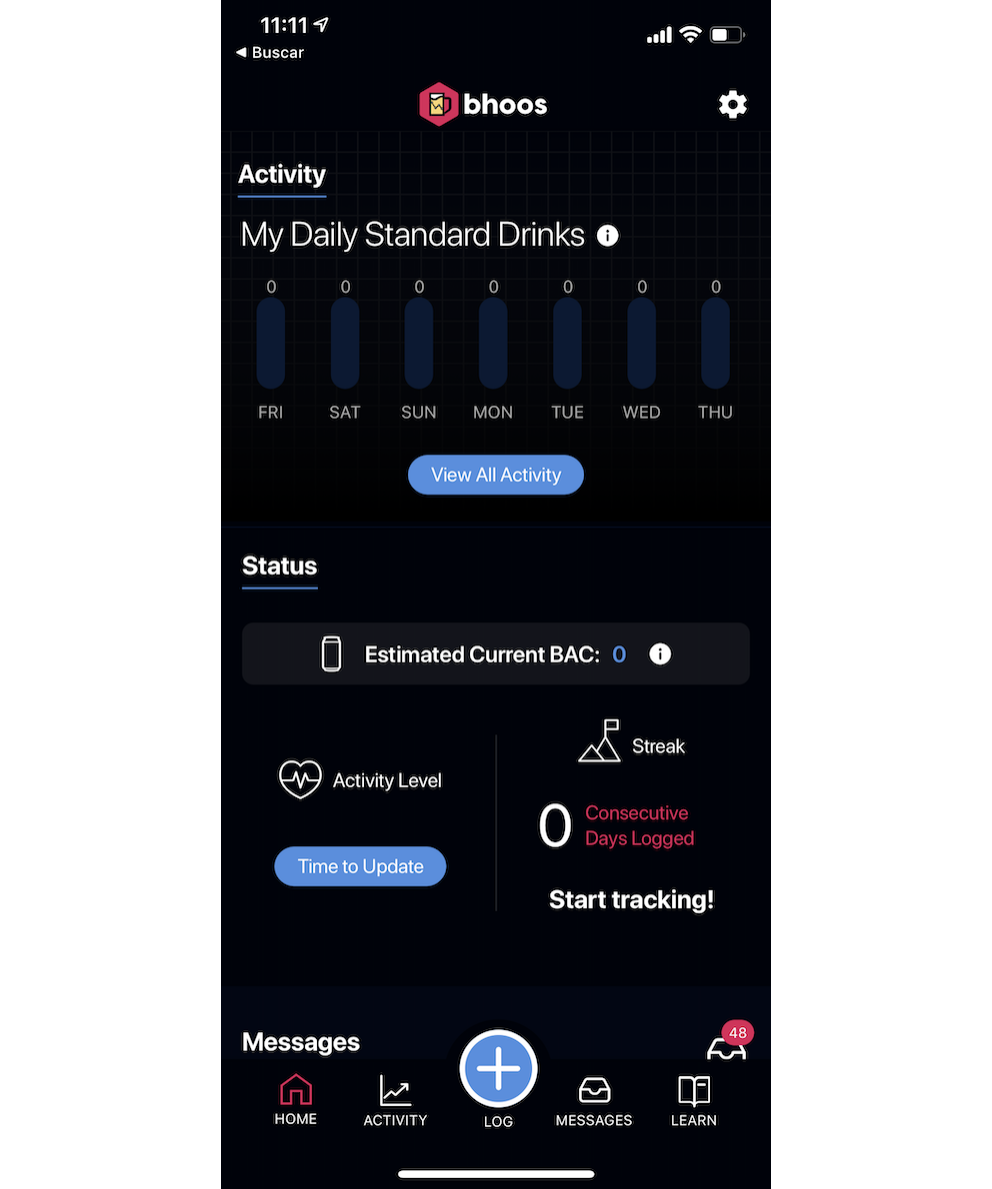
Home screen of the intervention (bhoos app). BAC: blood alcohol content.

**Figure 2:**
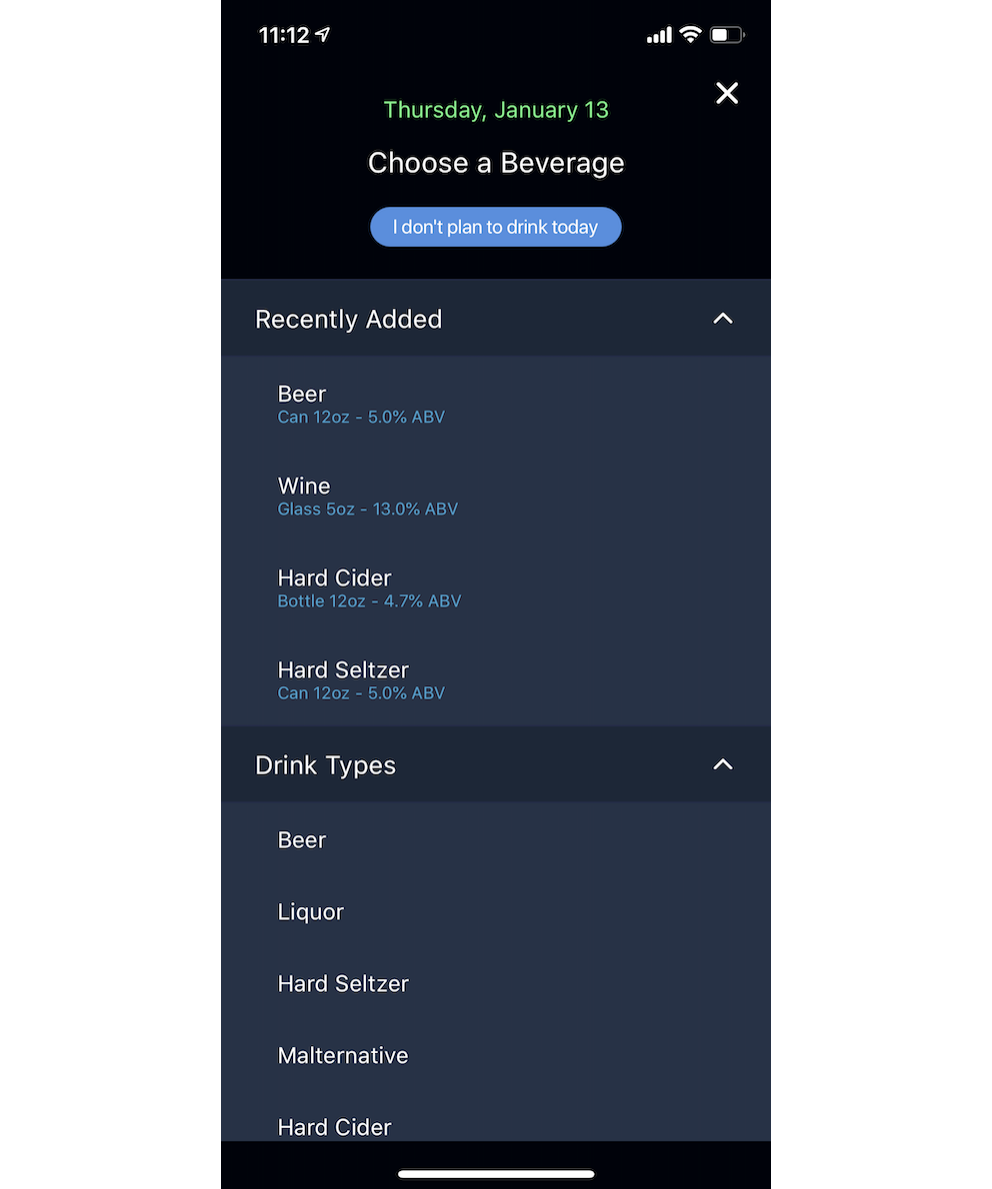
Drink tracker of the intervention (bhoos app).

**Figure 3:**
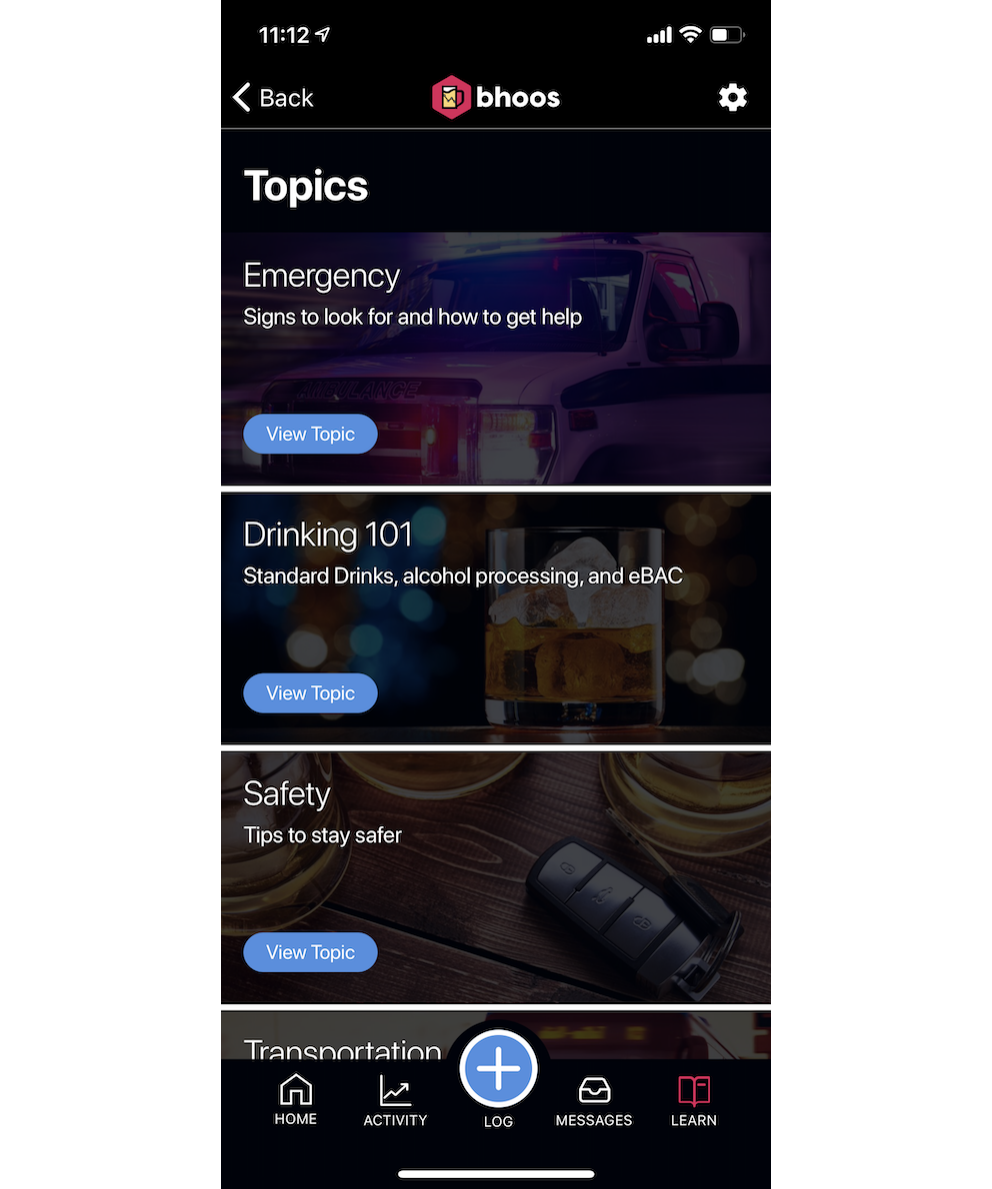
Learn topics of the intervention (bhoos app). eBAC: estimated blood alcohol content.

**Figure 4:**
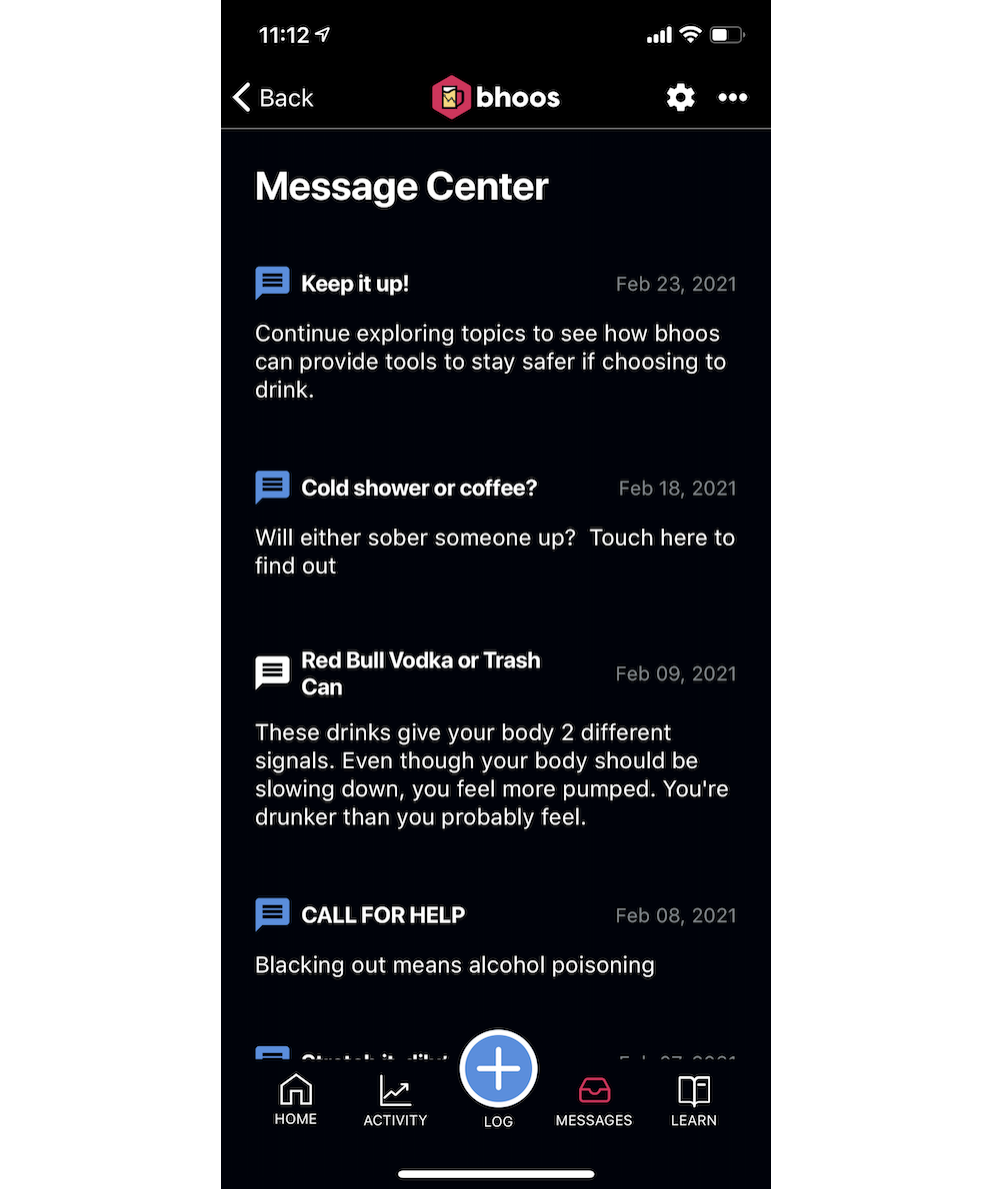
Message center containing student testimonials of the intervention (bhoos app).

Study team members created a draft prototype app based on prior experience in mobile app development and prior research on alcohol intervention development. Previous research indicates the benefits of self-monitoring for behavior change [18,19]. Logging risky behaviors, such as drinking, helps reduce those behaviors [14], leading us to include a drink tracker. To boost engagement, the app included personalized encouragement [20,21] to users who tracked several days in a row (streak). We used push notifications [22] to provide safety messages during heavy drinking episodes to alert the user of potential danger and provide recommended actions. In-app messaging, called the *message center* [22], provided *testimonials* from real students about drinking situations. To appeal to college student users’ needs, we added a secondary behavior (hydration or sleep) to track that could prompt users to engage with the app even when not drinking alcohol to keep users engaged [23]. The settings feature within the app allowed users to turn off push notifications and adjust their weight to reflect a more accurate eBAC. Once the prototype app was developed, 2 classes of undergraduate and graduate students in a mobile engineering class provided initial feedback on the draft app, and some features were slightly redesigned. Thus, the final prototyped app contained eight features and a home screen (Figure 1): alcoholic drink tracker (Figure 2); additional behavior tracker (hydration or sleep); self-monitoring feedback of days tracked; push messages about drinking and safety; eBAC calculator that provided an eBAC based on weight, sex, and timing or specific drinks logged; learning topics (Figure 3); a message center containing student testimonials (Figure 4); and settings.

### Study Design

The study included a single 1-hour qualitative interview of each undergraduate student participant using standardized think-aloud methods [24], described below. This study was approved by the Social and Behavioral Science Institutional Review Board of the University of Virginia (protocol 3282).

### Eligibility Criteria

To be eligible, a study applicant had to be (1) a student at the university, (2) able to read and speak English, and (3) aged ≥18 years. Participants reviewed an information form before completing any study activities and provided verbal consent that was audio recorded for documentation. The first round of interviews consisted of students who were not members of Greek organizations or student athletes. Participants in the second round included students who were fraternity and sorority members and student athletes. These groups tested the prototype app separately because previous research has shown that student athletes or who are in Greek organizations are at a higher risk for dangerous drinking episodes than their non-Greek peers [25-29], and we sought to gather feedback from groups who may have different alcohol use patterns.

### Procedures

#### Recruitment

Research coordinators and undergraduate research assistants (RAs) at the Center for Behavioral Health and Technology and the Center for Addiction and Prevention Research conducted the study. Participants were 10 university students aged 18 to 30 years. The sample was small because of disruption in recruitment as a result of the beginning of the COVID-19 pandemic. Participants were recruited from undergraduate and graduate classes, via flyers posted on bulletin boards in academic buildings around campus, via digital flyers posted on social media (eg, *GroupMe* and *Facebook* class pages), and by word-of-mouth referral from students working at the Center for Behavioral Health and Technology. The flyers contained a scannable QR code that linked directly to a Qualtrics interest form. The interest form collected applicant contact information, year in university, age, availability, and willingness to be contacted.

#### Think-Aloud Procedure

The *think-aloud* process is widely used for app development, as a common usability tool [24,30]. However, unlike a formal qualitative coding process that involves a thematic analysis to discover main findings [31], we used the think-aloud process to gather feedback on the app. Researchers asked participants to use a prototype mobile app, prompting them to use specific features while continuously speaking their thoughts and actions out loud [24]. Participants (N_total_=10, n_in-person_=5, n_zoom_=5) reviewed an web-based mock-up of the app with hot spots that responded to taps to mimic app functionality. Participants used a QR code they scanned on their personal smartphones to access the app mock-up on *InVision*, a collaborative digital design platform [32]. Participants navigated through the prototyped app, speaking aloud what they were doing and noticing, and providing feedback about app features, navigation, and perceived utility as they used them. When necessary, researchers reminded study participants to maintain a running commentary of their thoughts and noted the student’s navigation process as they used all aspects of the app. Researchers audio recorded the interviews and took thorough notes on a Word document while the participant was providing feedback. After completing the think-aloud procedure, participants received a US $10 Amazon gift card.

#### Modifications to the Protocol Due to COVID-19

The study was conducted in 2 rounds between March 3 and April 17, 2020. The first round of interviews occurred in person from March 3 to 6, 2020, that is, in early 2020, before the emerging COVID-19 pandemic required pausing in-person research. For in-person think-alouds, RAs watched the participants use the prototype while standing nearby and recorded their observations of student interactions and spoken thoughts of specific app pages on a note-taking sheet populated with the app screenshots. We developed a note-taking framework with screenshots of each page of the app grouped by app location (home, activity, log, message, and learn). Each screenshot provided space for think-aloud notes. RAs audio recorded the session using a handheld audio recorder or phone to supplement their written notes; when possible, >1 person in the room took notes or listened to the audio tape and took notes [30]. RAs reviewed the recordings after the trials and added further notes that were missing from the original notes to the summary document, which was finalized for review.

#### Between Rounds: App Changes Made in Response to Feedback

After the first round, the researchers debriefed the developers and investigators. Feedback showed that some labeling was unclear and users wanted more options for personalization and wanted colors and iconography to make data entry easier and more intuitive. Investigators and developers discussed which changes were needed immediately and prioritized changes (eg, adding and removing testimonials from the message center was prioritized lower than improving the labels and usability of the drink tracker). The development team fixed the functionality and clarity concerns reported in the round 1 trials.

Changed features included the eBAC, alternate behavior tracking, and calendar day view on the activity home screen. The eBAC was perceived as too small in the original mock-ups and was made more prominent on the dashboard. In addition, its colors were changed to correspond to which *zone* of drinking risk the user is in, based on the eBAC (eg, *golden zone*) [33]. Hydration was intended to be an alternative or secondary behavior that students could track, so they could track something other than alcoholic beverages. However, testing showed that the concept of hydration was confusing to users because they did not know if they should enter it once at the end of the day or update it multiple times throughout the day. The team switched to an alternate behavior that can be rated based on the previous full day, such as sleep quality. In addition, the calendar day view became more and more informative. For example, in the first version of the app, the day view showed only the number of drinks, but developers added the ability to record each drink, to compute a standard drink total, and to duplicate or mark drinks as a favorite and added the alternative behavior tracking feature to the calendar day view. Within the weekly and monthly views of behavioral metrics calculated by the app, graphs evolved to show metrics for the alternate behavior over those periods.

Students were sent home from the university in mid-March 2020, and in-person research was temporarily halted. To adapt to the situation, the second round of interviews between April 9 and 17, 2020, were conducted via Zoom video calls and used the second-round version of the app. The interviewer shared the screen, showing each of the app features one at a time. The participants installed the *InVision* mock-up on their personal device to enable navigation as if on an app and navigated simultaneously as the RA showed each screen. The note-taking and recording procedures were unchanged except for conducting them remotely. Information saturation was reached during the second round of interviews. Second-round participants’ comments and ideas were redundant with data from the first round. Major themes emerged naturally as the researchers summarized their notes. The research team determined that coding using qualitative software was not necessary.

### Data Review and Analysis

Once all of the notes were captured, the RAs deleted the audio recording. Researchers reviewed the notes and presented the major findings to the app development team for discussion and identification of features and functions to be modified. Reporting was in aggregate only (ie, “5 of the 20 participants said x, while 12 said y and 3 said z”). The app development team continuously iterated and updated the look and feel of the mock-up based on the feedback. Data included audio recordings of the think-aloud protocol process, notes taken by researchers observing participants, and usability questionnaire responses. All participants were assigned a unique ID unrelated to their identity. Stored data were linked only to this unique ID. All data were stored behind the Health Insurance Portability and Accountability Act firewall of an institutional review board-approved School of Medicine highly secure data drive.

## Results

### Overview

Demographic characteristics of the overall study sample and by condition are presented in Table 1. Participants were mostly women (7/10, 70%) and aged 20 years (5/10, 50%), and 80% (8/10) were enrolled in the most populous school within the university, containing departments in the Arts and Sciences.

**Table 1:** Sociodemographic characteristics of participants.

Characteristics		Total (N=10), n (%)	Round 1 students (n=5), n (%)	Round 2 students (n=5), n (%)
**Gender**				
	Male	3 (30)	2 (40)	1 (20)
	Female	7 (70)	3 (60)	4 (80)
**Age (years)**				
	18	1 (10)	1 (20)	0 (0)
	19	2 (20)	0 (0)	2 (40)
	20	5 (50)	3 (60)	2 (40)
	21	1 (10)	0 (0)	1 (20)
	22	1 (10)	1 (20)	0 (0)
**Year in university**				
	1	3 (30)	1 (20)	2 (40)
	2	3 (30)	1 (20)	2 (40)
	3	3 (30)	2 (40)	1 (20)
	4	1 (10)	1 (20)	0 (0)
**School**				
	Arts and Sciences	8 (80)	4 (80)	4 (80)
	Engineering	1 (10)	1 (20)	0 (0)
	Undergraduate Business	1 (10)	0 (0)	1 (20)

### Round 1

The feedback provided by the participants *in person* during round 1 is presented in Table 2. These students provided feedback on the eight essential features of the app: drink tracker, alternate behavior tracker (hydration or sleep), days tracked, messages, settings, eBAC calculator, learn topics, and a message center containing student testimonials. During the first round, RAs asked participants to focus on usability, rather than aesthetics, of the app. Participants expressed confusion about the purpose of the features and provided useful feedback about how they were displayed. For example, the drink tracker had drink choices listed that the students wanted to rename (eg, they recommended renaming *seltzer* or *cider*, to *hard seltzer* and *hard cider*, respectively, to ensure that future users would log alcoholic versions of these drinks) and indicated that such changes would increase utility and relevance. Participants reported that they would prefer having brand name examples (eg, *Corona*, *Coors Lite*, and *White Claw*) for each of the choices (ie, beer, liquor, seltzer, mixed drink, cider, and wine) to help clarify the different types of alcoholic drinks, again increasing utility and relevance as well as appeal. They reported that this would help future student users accurately track their drinks and that they believed many students do not know the alcohol by volume percentages for typical drinks.

**Table 2:** Round 1: summary of responses about features in prototype app (n=5).

Feature and feedback provided		Value, n (%)
**Drink tracker**		
	*Seltzer* and *cider* renamed as *hard seltzer* and *hard cider*, respectively, to convey that these are alcoholic drinks	3 (60)
	Want examples of each alcoholic drink (eg, for beer, to have options such as Corona, Modelo, and PBR^a^) not only for clarification but to select since not everyone will know the alcohol by volume percentage	3 (60)
	Confusion around the green fill bars on the dashboard or home screen; unclear whether this was displaying a proportion of drinks consumed to a limit that was set	2 (40)
	Confusion as to whether there’s an option to track zero drinks	2 (40)
	Option to log a *recent drink* for ease of use rather than having to tediously enter the information for the same drink again	2 (40)
**Hydration tracker**		
	Thinks the metric is too subjective; the scale is too ambiguous (eg, not sure what the difference, for example, in ounces of water, that would be categorized as a *1* or *2*)	5 (100)
	Feature not necessarily useful	2 (40)
	Does not understand the purpose of this feature	1 (20)
**Days tracked**		
	Metric unclear for measuring days tracked (eg, 10 days tracked since not tracking or 10 days since downloading the app?)	2 (40)
	Display next to the drink tracker, rather than the hydration tracker	2 (40)
	Add an option to share this with other app users to compare with friends	1 (20)
**Messages**		
	Confusion around the *thumbs up or down* buttons in the header	4 (80)
	Confused as to what this feature is entirely	4 (80)
	Preference for having messages be set at the dock along the bottom with the home, activity, and learn buttons	1 (20)
**Settings**		
	Profile data (ie, biological sex, age, and weight) accessible here	4 (80)
	Option to decide types of messages are received here	3 (60)
	Default setting have push notifications set to *off*	2 (40)
	App FAQs^b^ would fit better if relocated here	1 (20)
**eBAC^c^ calculator**		
	Display statistics of *current BAC*^d^ and *maximum BAC level*	4 (80)
	Remove *Time until legal limit (0.08%)* in case it might be misleading or encourage driving while intoxicated	2 (40)
	Option to see an *Average BAC* or drinking trends over time	2 (40)
	Should be the first thing you see when you open the app	2 (40)
**Learn topics**		
	Information should be condensed so no scrolling is required	3 (60)
	Confusion on the difference between this and other features in the app	3 (60)
	Do not think that this will be a used	2 (60)
**Message center with testimonials**		
	Pushing too much content with this feature; this is no different from previous features	3 (60)
	Option to control the frequency of messages and notifications	3 (60)
	Would like the *thumbs up or down* button moved here so they could indicate preference on types of messages and rate the content for future app updates	2 (40)

^a^PBR: Pabst Blue Ribbon.

^b^FAQ: frequently asked question.

^c^eBAC: estimated blood alcohol content.

^d^BAC: blood alcohol content.

Participants also provided feedback on whether they were interested in and would use certain features. For example, students were not interested in a hydration tracker feature and considered its utility and appeal low. The hydration tracker asks students to rate how hydrated they are on a scale of 1 to 5. Participants expressed confusion about the hydration tracking as it was not as specific as the (alcoholic) drink tracker. Students questioned how the hydration tracker related to the app, failing to see any connection between hydration and drinking alcohol. Thus, the hydration tracker showed low relevance.

### Round 2

Participants in round 2 met with the RAs on the web using video meeting technology, but other than this, all other procedures remained similar. In the second round of think-aloud testing, RAs guided participants to focus on the look and feel (eg, aesthetics and design) more than the functionality of the app. For example, the app’s default setting is in *dark mode*, with a black background; a participant expressed preference for a *light mode* option to increase its appeal. In addition, participants expressed a preference for symbols for sex rather than the terms *male* and *female* for personal data entry screens. Participant feedback in round 2 is presented in Table 3. Overall, participants expressed interest in several of the features of the app, but not in others. The look and feel of the app were reported to be important to the students. Student navigation across app features indicated that the improved look and feel helped guide them to the features they were most interested in using.

**Table 3:** Round 2: look and feel and features in prototype app (n=5).

Feature and feedback provided		Value, n (%)
**Drink tracker**		
	Likes the log button and is easily accessible	3 (60)
	Likes that there is a *recently added* shortcut for logging recent drinks	2 (40)
	Does not think that students will use the feature; thinks that students would forget to log drinks on a night out	1 (20)
**Sleep tracker**		
	Does not understand the connection or link between sleep and drinking	5 (100)
	Does not believe the feature be used	2 (40)
**Days tracked**		
	Do not believe that this feature is necessary	2 (40)
	Rather than days tracked, would prefer a tracking streak of days not drinking	1 (20)
**Messages**		
	Preference for having messages be set at the dock along the bottom with the home, activity, and learn buttons	2 (40)
	Likes the *unread* symbol that alerts the user to open messages	1 (20)
**Settings**		
	Option for *light mode* or a white interface	1 (20)
	Option for specific *push* notification	1 (20)
	Option to share *data* with friends to boost engagement	1 (20)
**eBAC^a^ calculator**		
	Increase in font size for the BAC^b^ statistics	2 (40)
	Prefer the sex symbols to change to words on home screen	1 (20)
**Learn topics**		
	Believed this section to be visually appealing	2 (40)
	Add images to help visualize the concept of *standard drinks*	1 (20)
**Message center with testimonials**		
	Seems to be helpful	3 (60)
	Would prefer graduating class over age (eg, Jessica, Class of 2021, UVA^c^ activities, instead of—Jessica, 21, UVA activities)	2 (40)
	Testimonials are too long and too dense; would prefer bullet points over long sentences	2 (40)

^a^eBAC: estimated blood alcohol content.

^b^BAC: blood alcohol content.

^c^UVA: University of Virginia.

## Discussion

### Principal Findings

The objective of this study is to assess the appeal, relevance, and perceived utility of a draft mobile app for college students that could intervene at or around the time of alcohol use. We sought to determine if students would want and use the app and if it would be perceived as useful and engaging. Participants reported that minor changes to the functionality of the initial versions of the app would improve the usability of the app (eg, having examples of each alcoholic drink for clarification and stating whether they believe certain features would be used or not). In addition, participants reported that minor changes to the aesthetics would increase the usability (eg, incorporating the logo colors throughout the app to make it more vibrant and having an option to turn off push notifications). The changes were made between rounds of think-aloud user testing to enact modifications based on user feedback.

This process was an iterative step within a series of formative studies to create and improve an app to reduce college binge drinking. Feedback provided by college students assisted the investigators and development team in designing features that were useful and appealing. The purpose was to test usability, and student feedback helped the team modify the app so that it would perform as students hoped in *real-life* scenarios to address binge drinking and alcohol consumption. This process was designed as a feedback loop beginning with the *science* that drafted features about learning what a standard drink is, self-monitoring tools with immediate and longer-term feedback about risks when drinking, options to get help in an emergency, and vetted information about sex differences in alcohol processing in the body. The features were conceptualized and then mocked up by developer team members with expertise in user experience and user interface design). Finally, the team obtained qualitative feedback on relevance and usability from students regarding the aesthetics and appeal of the app and its ability to engage them.

### Limitations: Modifications Due to COVID-19

Although the practical insights from students about the app proved valuable and guided us to make important app modifications, there are several limitations to be considered. Because of COVID-19, students had to participate in the second round of think-aloud trials virtually over Zoom using an adapted protocol. Although participants navigated through the app with ease, the researchers could not always see the participants’ phone screen and sometimes had difficulty determining which features the participants were commenting on. RAs asked what feature the participant was viewing and where in the app they were navigating to and prompted the participant to turn their phone screen toward the camera. These moments of clarification seemed to interrupt the natural rhythm and flow of the think-aloud format and may have resulted in reduced information. In person, the researcher could easily see the participant’s phone screen without prompting, which allowed for the conversation to flow uninterrupted while the RA could take notes more inconspicuously. During round 1, the participants’ comments were prominent in the conversation, whereas researchers listened and asked fewer questions. Conversely, researchers asked more questions during round 2, whereas participants provided less feedback, and their comments were either positive or more superficial in nature.

Previous studies have used apps to help reduce dangerous drinking episodes among college students. However, these apps did not have or include features that tailor to individual personalized feedback such as logging drinks and providing an eBAC [34-36]. One of these previous interventions has justified the use of a digital platform as users did indicate that it offered help and was easy to use [36]. Other alcohol app interventions have reported to significantly reduce drinking [34,35].

### Future Research

Data and conclusions from this study will be synthesized with information gained from other qualitative and quantitative formative work, to determine the final features to be tested. We plan a pilot randomized trial to assess the use and impact of the app on drinking behavior over several months; we plan to conduct a prospective brief observational and feasibility study with about 255 students to (1) measure student demographics, attitudes, experiences, and behaviors related to drinking; (2) evaluate the acceptability, feasibility, relevance, and actual use of the app over the intervention period among students; (3) measure student attitudes, experiences, and behaviors related to drinking in a final survey; (4) determine the usability of the app and desired improvements using short interviews after about 4 weeks of app use; and (5) test the impact of incentives on app use to provide crucial information for implementation of a larger subsequent trial. If the pilot of this mobile app intervention shows acceptable use and reporting of frequency and volume of binge drinking, we will consider testing it further for impact on drinking behaviors.

### Conclusions

Student feedback clearly indicated preferences for the eBAC calculator and days tracked features. In contrast, student feedback showed a dislike of or lower interest in using the water or hydration tracker, learn topics, and messages or testimonials. On the basis of student ratings of appeal, relevance, and utility, we revised the app, and the finalized app included the drink tracker, days tracked, eBAC calculator with an eBAC level, sleep quality tracker, learn topics, message center, and testimonials. By asking student participants to navigate through, and comment on, the app’s features using standard think-aloud procedures, the researchers gained real time perspective on the appeal, relevance, and utility of the app that could be translated into a refined version of the app.

## Abbreviations

eBAC: estimated blood alcohol content
RA: research assistant
